# Agro-ecological suitability assessment of Chinese Medicinal Yam under future climate change

**DOI:** 10.1007/s10653-019-00437-w

**Published:** 2019-10-15

**Authors:** Dongli Fan, Honglin Zhong, Biao Hu, Zhan Tian, Laixiang Sun, Günther Fischer, Xiangyi Wang, Zhiyu Jiang

**Affiliations:** 1 Shanghai Institute of Technology, Shanghai, 201418 China; 2 Department of Geographical Sciences, University of Maryland, College Park, MD 20742 USA; 3 School of Environmental Science and Engineering, Southern University of Science and Technology, Shenzhen, 518055 China; 4 School of Finance and Management, SOAS University of London, Russel Square, London, WC1H 0XG UK; 5 International Institute for Applied Systems Analysis (IIASA), 2361 Laxenburg, Austria

**Keywords:** Chinese medicinal yam, AEZ model, Climate change, Suitable planting area

## Abstract

Chinese Medicinal Yam (CMY) has been prescribed as medicinal food for thousand years in China by Traditional Chinese Medicine (TCM) practitioners. Its medical benefits include nourishing the stomach and spleen to improve digestion, replenishing lung and kidney, etc., according to the TCM literature. As living standard rises and public health awareness improves in recent years, the potential medicinal benefits of CMY have attracted increasing attention in China. It has been found that the observed climate change in last several decades, together with the change in economic structure, has driven significant shift in the pattern of the traditional CMY planting areas. To identify suitable planting area for CMY in the near future is critical for ensuring the quality and supply quantity of CMY, guiding the layout of CMY industry, and safeguarding the sustainable development of CMY resources for public health. In this study, we first collect 30-year records of CMY varieties and their corresponding phenology and agro-meteorological observations. We then consolidate these data and use them to enrich and update the eco-physiological parameters of CMY in the agro-ecological zone (AEZ) model. The updated CMY varieties and AEZ model are validated using the historical planting area and production under observed climate conditions. After the successful validation, we use the updated AEZ model to simulate the potential yield of CMY and identify the suitable planting regions under future climate projections in China. This study shows that regions with high ecological similarity to the genuine and core producing areas of CMY mainly distribute in eastern Henan, southeastern Hebei, and western Shandong. The climate suitability of these areas will be improved due to global warming in the next 50 years, and therefore, they will continue to be the most suitable CMY planting regions.

## Introduction

Yams (*Dioscorea* spp.) are a multispecies crop planted in over 50 countries. Although in comparison with other root and tuber crops, the production of yams is expensive because of high planting and labor costs, a long growing season, and low yield per hectare, they hold cultural and social importance and have preferred organoleptic properties (Price et al. 2016). In China, yams have been widely accepted for the reasons of both organoleptic properties and potential medicinal benefits as prescribed by the Traditional Chinese Medicine. According to our surveys of the main Chinese herbal medicine markets and the planting bases of Chinese Medicinal Yams (CMY) in China, about 10–20% of yam output are used as medicinal food every year. In China, the annual planting area of medicinal yams is about 2,000 km^2^ and the amount of yams used for medicinal is about 20,000 tons per year. CMY is represented by Taigu Yam and Huai Yam in the north of Henan Province and the south-central part of Shanxi Province. According to the literature of Traditional Chinese Medicine, CMY has high medicinal and nourishing value to the human health (Commission 2015). It can be used for various treatments of spleen deficiency and chronic diarrhea, chronic enteritis, lung deficiency and cough, chronic gastritis, diabetes, nocturnal emission, enuresis, and underlying embolism (Cheng and Li 2010; Wang et al. 2006). As an increasing number of people pay more attention to health preserving, the demand for CMY on Chinese herbal medicine market has kept increasing in recent years.

Climate change has a great impact on the crop phenology, so does the formation of authentic medicinal materials in CMY. Many studies report (Guo et al. 2005; Xu et al. 2009; Li et al. 2019) that the authenticity is the core symbol of the quality of Chinese medicinal materials. The specific locations of medicinal yams have been a manifestation of their obvious geographical characteristics, which are closely related to their demands for special climate, soil, and other ecological conditions (Wang et al. 2010). The climate conditions are important factors influencing the yield and quality of yams in parallel to soil factors (Zhou et al. 2006; Liu et al. 2007). Recent studies have found that variation in climatic factors and complex topography makes the quality of yams different (Wu 2014; Li et al. 2009).

One of the most reliable and effective methods to determine the suitable planting area of different crop is to conduct field experiments. However, it is difficult to carry out field experiments in large scale over a number of years due to the constraints of manpower, material resources, and duration of observations over several growth cycles for a number of years. As a consequence, crop models have become one of the most powerful tools in agricultural science research, in which the key mechanisms of crop growth are rigorously incorporated and the role played by crop cultivation management has been increasingly recognized. The field of their application is expanding. For example, Tian et al. (2014) developed a model coupling and fusion method to assess the impact of future climate change on crop growth and yield. Srivastava et al. (2012a, b, 2016) used the EPIC crop growth model to simulate the impact of fallow availability on yam yield in West Asian based on historical data and to estimate the climate change impact on yam yield in the same region. Marcos et al. (2011) employed the CropSystVB model to assess the effect of planting date on yam growth and yield at the Experimental Station of Duclos in French Antilles. However, current studies focus on the production quantity of yams and its change, being unable to identify the medical quality variation associated with climate change, soil, and geographical conditions. Moreover, Raymundo et al. (2014) argued that the ability of both the CropSystVB-Yam and EPIC-Yam models in climate change impact assessment is limited mainly owing to the lack of calibrations with modern cultivars across agro-climatic zones.

Chen (2011) employed the Geographic Information System (GIS)-based statistical techniques and historical data to make the digital suitability zoning of various Chinese medicinal plants in China. However, such studies are unable to estimate the impact of future climate change on CMY production. Hu et al. (2018a, b) evaluated the suitable regionalization of CMY based on GIS technique and similarity comparison of thirteen ecological indicators that were selected on the base of literature review and experts’ opinions to represent the key geo-authentic features of CMY. These indicators are aggregate in nature, and such index-based similarity comparisons do not involve rigorous crop growth modeling at all. In particular, the indicators include only four soil indicators (soil type, PH, organic carbon, and cation exchange capacity), which would be too brief to effectively represent the soil-sensitive authentic features of CMY.

In this study, we employ the agro-ecological zone (AEZ) modeling tool, with the input of the climate projection results from several global and regional climate models under the Representative Concentration Pathways (RCPs), to simulate the suitable regionalization of medicinal yam in the future, and applied the crop model to the study of suitable planting areas of CMY. We improved the AEZ model by expressing the quality and medicinal properties of CMY as the varietal parameter information of CMY and transforming it into the physiological and ecological parameters of the AEZ model. We validate the updated CMY varieties and AEZ model using the historical planting area and production under the observed climate conditions. After the successful validation, we use the enriched AEZ model to simulate the potential yield of CMY and identify the suitable planting regions under future climate projections in China. Such a suitability assessment will provide useful information for decision makers and other stakeholders in the CMY industry to plan the layout of CMY industry, to ensure the quality and supply quantity of CMY, and to safeguard the sustainable development of CMY resources for public health in the future. In this way, our research fills in a very important gap in the literature of Chinese herbal medicine research.

## Study area and data

### Study area and observation sites

**Fig. 1:**
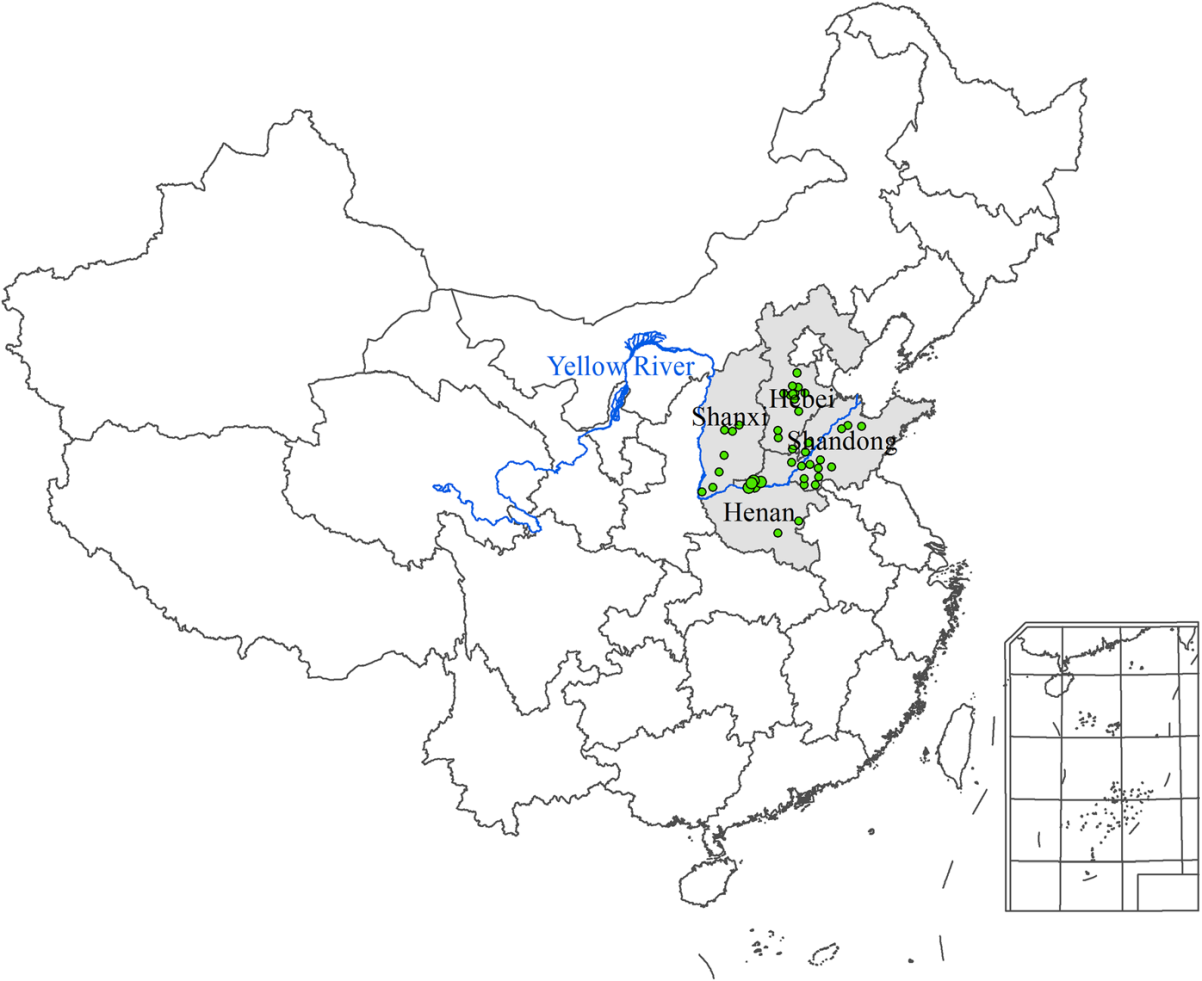
Location of the 42 representative sites in the genuine production area of CMY (all green dots) and the five sites producing the highest quality CMY (larger dots)

We obtained the data on genuine producing areas of CMY from both internet search and fieldwork based investigations of the Chinese herbal medicine markets and the officially assigned CMY planting bases.^1^ The genuine producing areas refer to the traditional and Geo-authentic producing area where CMY has been planted since ancient times. We identified 42 sites as the representative sites of the genuine yam areas for verifying our AEZ-China Yam simulation model. Of these 42 sites, we further identified five sites which have been consistently and widely acknowledged as the best locations for high-quality medical yam production. For these five sites, consistent agro-meteorological records for yam production are also available (Fig. 1 and Table 1). Figure 1 shows that the CMY planting area is mainly located along the Yellow River Basin plains in North China.

**Table 1:** Key agro-meteorological indicators of CMY in the key growth periods at the five best CMY producing sites. *Sources*: Liu et al. (2007), Wang et al. (2010) and our own fieldwork

Growth cycle	Dates	Optimum temperature (°C)	Lower boundary of minimum temperature (°C)	Upper boundary of maximum temperature (°C)	Optimum moisture content (%)	Upper limit of moisture content (%)	Rhizome percentage of total growth period (%)
Sowing period	Late March to mid-April	20	10	–	–	–	–
Emergence period	Early April to mid-May	20	12	–	15–18	25	–
Seedling period	Early to mid-May	15–20	0	–	–	–	**–**
Vigorous growth period	Mid-May to mid-June	25–28	10	40	16–20	–	< 2
Bud period	Mid-to-late June	25–28	–	35	–	–	< 2
Tuber formation period	Early and mid of July to Sep. 10	20–24	3	24	18–20	22	70–90
Mature period	Sep. 10 to the end of Oct	–	–	–	–	–	10–15

– Stands for data that are not needed in this study.

### Data

#### Observation records of yam growth cycle in the CMY regions

Crop cultivar information in the AEZ model includes crop eco-physiological parameters (harvest index, maximum leaf area index, maximum rate of photosynthesis, etc.), crop management, utilization of main products, crop residues, and by-products (Fischer et al. 2002, 2012). The first key objective of this research is to enrich and update these crop eco-physiological parameters in the AEZ model. Table 1 shows the agro-meteorological indicators of CMY in different growth periods of the five best sites. These data are used as reference for updating eco-physiological parameters in the AEZ model. We improved the optimal conditions in the AEZ model based on the yam variety parameters of the five sites. And we improved the range conditions in the AEZ model based on the yam variety parameters of all the 42 sites.

#### Observed climate data and future climate projections

The baseline climate data (1981–2010) are from the historically observed daily climate dataset provided by China Meteorological Data Center, including temperatures, precipitation, relative humidity, wet-day frequency, sunshine duration, and wind speed at 10 m height. All the input data are resampled to a spatial resolution of 1 km using ArcGIS.

The future climate projections are obtained from Inter-Sectoral Impact Model Intercomparison Project (ISI-MIP) (Warszawski et al. 2014), which is a community-driven modeling effort with the goal of providing cross-sectoral global impact assessments. Projections of future climate were obtained using five general circulation models (GCMs) from Coupled Model Intercomparison Project, Phase 5 (CMIP5), and the Representative Concentration Pathways (RCPs) for carbon emissions supported by the IPCC Fifth Assessment Report (AR5). The ISI-MIP models dataset selected in this study are GFDL-ESM2M, HadGEM2-ES, IPSL-CM5A-LR, MIROC-ESM-CHEM, and NorESM1-M.

#### Soil and land use

Soil data are extracted from the Harmonized World Soil Database (HWSD). The HWSD was developed by the Food and Agriculture Organization (FAO) and the International Institute for Applied Systems Analysis (IIASA) (Fao/Iiasa/Isric/Isscas/Jrc 2012). This dataset provides reliable and harmonized soil information at the grid cell level for the world, with a spatial resolution of 1 km for China. It provides detailed information on soil parameters, such as textural class, clay fraction, drainage rate, bulk density organic carbon, pH, and cation exchange capacity. The soil data are divided into topsoil (0–30 cm) and subsoil (30–100 cm). According to the soil environment in which Chinese Medicinal Yam grows, we choose the subsoil factor as the main object of analysis.

Land use data (spatial resolution of 100 m) in 2015 are downloaded from resource and environment data cloud platform of Chinese Academy of Sciences. Land use types include 6 primary types and 25 secondary types. Main land use types are cultivated land, forest land, grassland, water area, residential land, and unused land. The cultivated land data are further subdivided into four sub-types of mountains, hills, plains, and slopes larger than 25 degrees under both rainfed and irrigated conditions. The potential yields of CYM are simulated on the cultivated land for the suitability analysis.

## Methodology

### The AEZ model

The AEZ model used in this study was jointly developed by the Food and Agriculture Organization of the United Nations (UN-FAO) and the International Institute for Applied Systems Analysis (IIASA). It is a model mainly used for crop suitability assessment and production potential calculation. It has a well-acknowledged advantage in simulating crop growth across grid cells of a large region (Fischer et al. 2012). The AEZ model was used to evaluate the cereal production potential of cropland of individual countries in the 1990s. It has also been used to study the production potential of wheat, corn, rice, and many other crops in China. The basic principle of the model is to gradually correct the maximum biological yield of crops through limiting parameters such as cumulative temperature, moisture, soil suitability, and management methods and to simulate the maximum production potential of crops by irrigation and rainfed cultivation methods under high, medium, or low agricultural input conditions. The AEZ model can comprehensively consider the multiple climatic factors affecting crop growth. The required meteorological data are relatively easy to obtain. The required parameters and thresholds can also be adjusted according to actual conditions (Fischer et al. 2012). Because the model is rigorously logical and comprehensive, over the past 30 years, the model has been employed to examine many global and regional issues such as land carrying capacity, food production, crop suitability, soil erosion, and land degradation, especially in developing countries such as Bangladesh, Thailand, and China. In this study, we introduced the new CMY varieties into the AEZ model and then applied the enriched AEZ model to carry out suitability assessment of CMY under the projected climates in China. The AEZ model can quickly run across all grid cells in a large region and can easily take climate projection information into its agro-climate resource assessment module. We ran the AEZ model at each of the 1 km × 1 km grid cells across all cropland in China. As a consequence, all maps of simulation results are presented at the same resolution.

### The procedure of yam simulation

**Fig. 2:**
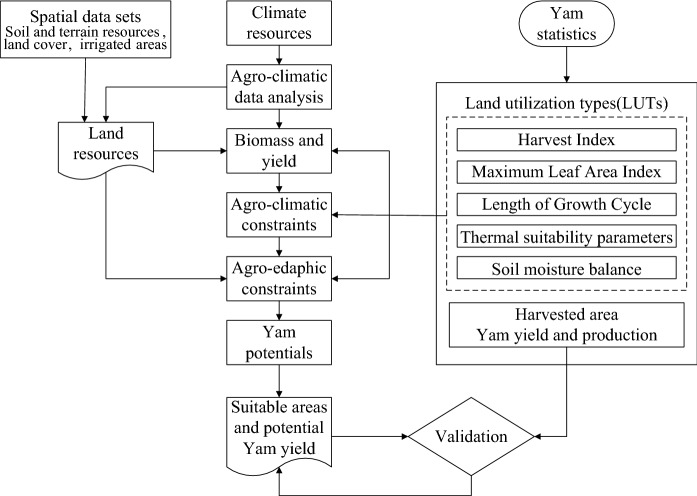
Framework for the AEZ-CMY modeling and validation in China

Figure 2 depicts the framework for the AEZ-CMY model validation and simulation in China. In order to introduce new CMY varieties into the AEZ model, we collected the information of key agro-meteorological indicators (Table 1), sowing date, growth period length, harvest index, leaf area index, yield, water demand, and soil attributes of CMY at the five best CMY producing sites. Considering that the focus of this research is on the medical quality of yam, we chose the best variety of CMY in the authentic production area to calibrate the AEZ model. We consider climate factors, soil factors, and field management practices of yam growth in the authentic production area for calibrate physiological and ecological parameters of CMY in the AEZ-CMY model. These input data for calibration include sowing date, length of growth period, accumulated temperature during growth period, harvest index, maximum leaf area index, yield, soil type, topography, and water demand.

In the AEZ model, temperature profile classes for CMY (Table 2) are catered for crop growth cycle duration in different classes of mean daily temperatures, in 5 °C intervals. These temperature profiles are matched with yam growth temperature requirements to generate either optimum match, sub-optimum match, or not suitable conditions (Fischer et al. 2012). The temperature profile and temperature sum of each phenological stage are calculated at each of the five best CMY producing site with observed daily meteorological and climate data.

**Table 2:** Definition of the temperature profile classes for Chinese Medicinal Yam

Average temperature (°C)	Growth cycle duration (days)	Temperature trend	
		Increasing	Decreasing
> 30	L1	L1a	L1b
25–30	L2	L2a	L2b
20–25	L3	L3a	L3b
15–20	L4	L4a	L4b
10–15	L5	L5a	L5b
5–10	L6	L6a	L6b

Table 3 reports the thermal suitability parameters of CMY in the AEZ-CMY model at each stage. According to our survey, the most suitable relative humidity range of CMY is 65.54–66.26%, and the secondary relative humidity range is 49–66.7%. Therefore, when simulating the growing suitability of yam, the optimum range for relative humidity is set between 55 and 70%, and the relative suitable interval is set from 45 to 75%. The most suitable cumulative sunshine hour of CMY is between 1300 and 1550 h. Therefore, the optimum cumulative sunshine hour is set to be greater than 1200 h, and the relative suitable sunshine condition is set between 1050 and 1200 h. A value smaller than 900 h means that the sunshine condition is not suitable for CMY growth.

**Table 3:** Thermal crop suitability parameters in the new LUTs

Optimal conditions	Range conditions
*L*6 = 0	*L*6 = 0
*L*5a < 0.0415*L	*L*5a < 0.125*L (tolerate 0.167)
*L*4a < 0.167*L	*L*4a < 0.250*L (tolerate 0.333)
*L*4b + *L*5b < 0.250*L	*L*4b + *L*5b < 0.333*L (tolerate 0.400)
*L*3b > 0.167*L	*L*3b > 0.083*L (tolerate > 0.010)
*L*1 < 0.083*L	*L*1 < 0.167*L (tolerate 0.250)
*L*1 + *L*2 + *L*2b < 0.500*L	*L*1 + *L*2 + *L*2b < 0.667*L (tolerate 0.833)
*L*1 + *L*2 + *L*2b > 0.167*L 55 < avg Relative Humidity < 70 Sunshine hour > 1200	*L*1 + *L*2 + *L*2b > 0.000*L 45 < avg Relative Humidity < 75 (tolerate 40–80) Sunshine hour > 1050 (tolerate 900)

*L* stands for the length of growth cycle (or growth period)

Table 4 presents the CMY variety parameters we have calibrated. The maximum length of growing cycle (LGC) is set at 210 days according to the observations at the five best CMY growing sites. Minimum temperature is defined as the threshold for counting the temperature requirements (temperature sum) during the LGC. According to Zhu et al. (2011), Wang et al. (2012), and Zhang et al. (2001), the harvest index (HI) of CMY is about 0.50. The lower and upper boundaries of accumulated heat units range (TS2) and optimum accumulated heat units (TS1) in Table 4 are set according to the accumulated temperature above 10 degrees (TS3). The mean, maximum, and minimum of TS3 were 4764, 5120, and 3829, respectively, at the five best CMY growing sites during the baseline climate of 1981–2010.

**Table 4:** New LUTs of Chinese Medicinal Yam added in AEZ model

NAME	CYA + CYB	TMN	TREF	HI	MLAI	YF%	TS2n	TS1n	TS1x	TS2x
YAM M1	0 + 180	10.0	23.0	0.50	3.00	0.51	3400	3750	4250	4500
YAM M2	0 + 195	10.0	22.5	0.50	3.00	0.54	3600	4000	4500	4750
YAM M3	0 + 210	10.0	22.0	0.50	3.00	0.57	4000	4400	4850	5200

Once the CMY variety parameters are calibrated, we first run the AEZ-CMY model under the baseline climate to verify the performance of the model. Once the model is verified, we run the AEZ-CMY model under the future climate scenarios to assess the impact of climate change on suitable areas and the best available yields of CMY. The left half of Fig. 2 depicts the major steps in the AEZ model.

## Results and discussion

### Validation of the AEZ-CMY model

**Fig. 3:**
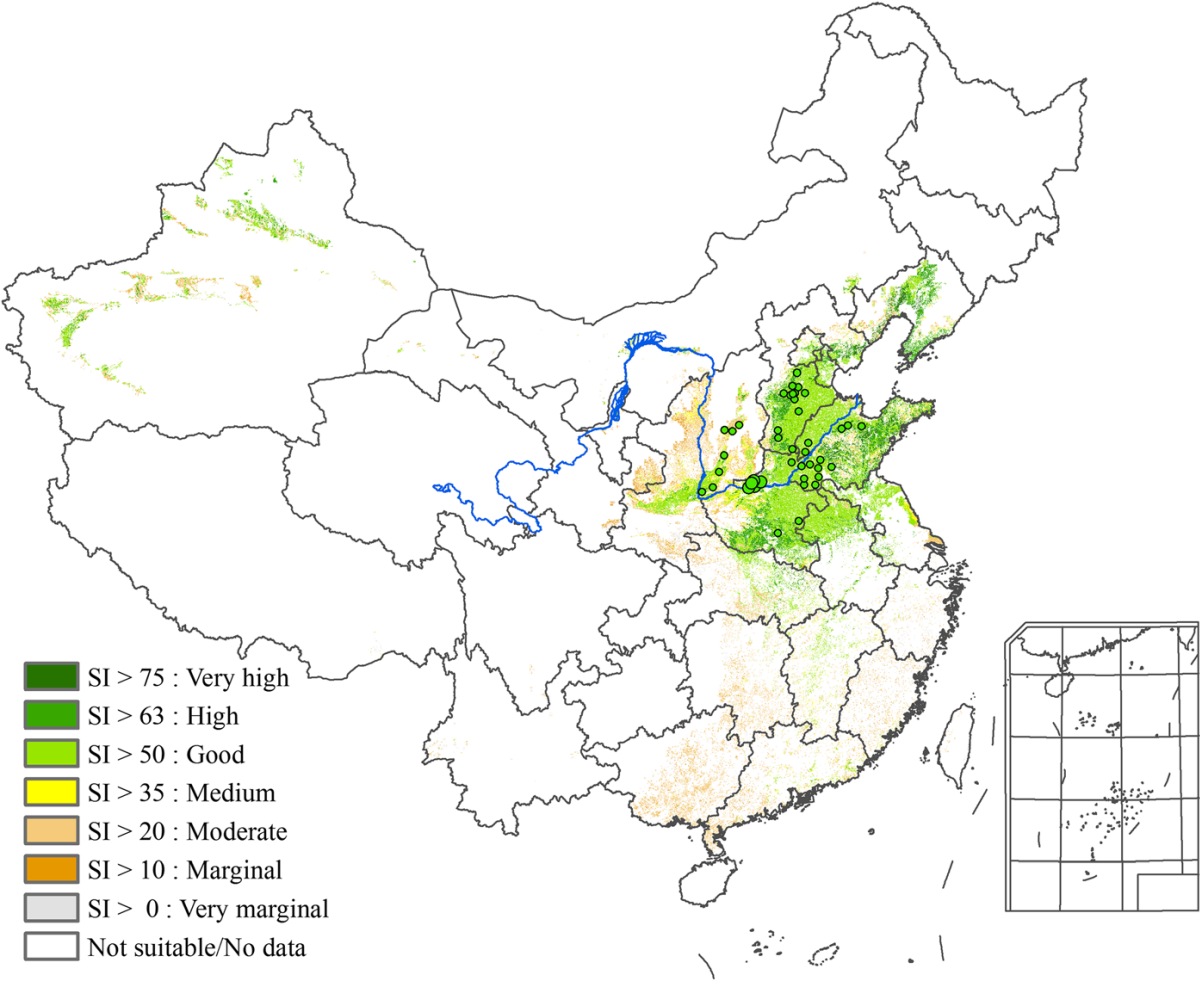
Model validation by suitability area

The special distributions of the simulated areas which have high agro-ecological similarity with the genuine producing areas of CMY under the baseline climate of 1981–2010 are shown in Fig. 3. The figure indicates that these areas are located in current CMY producing regions of eastern Henan, southeastern Hebei, and western Shandong. Figure 3 also shows that the distribution of the 42 sites coming from our survey matches well with the suitable planting areas of CMY produced by our AEZ-CMY simulation under the baseline climate.

**Fig. 4:**
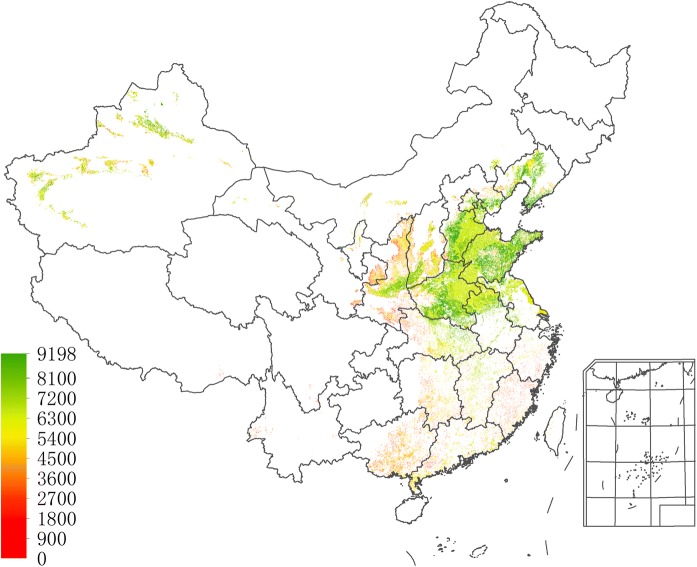
Model validation by the best attainable yield (kg DM/ha)

The simulation results of the best attainable CMY yield under the historical climatic conditions of 1981–2010 are shown in Fig. 4. It can be seen from Fig. 4 that the dry matter (DM) values of the best attainable yield are mostly concentrated in the range of 3,000–7,500 kg per hectare, which imply fresh weights of 10,000–25,000 kg per hectare, being well in line with the range of 15,000–22,500 kg fresh weight per hectare as observed in current main producing areas of CMY.

Figures 3 and 4 together provide a strong validation of our AEZ-CMY model.

### Impact of climate change on suitable areas for CMY cultivation

**Fig. 5:**
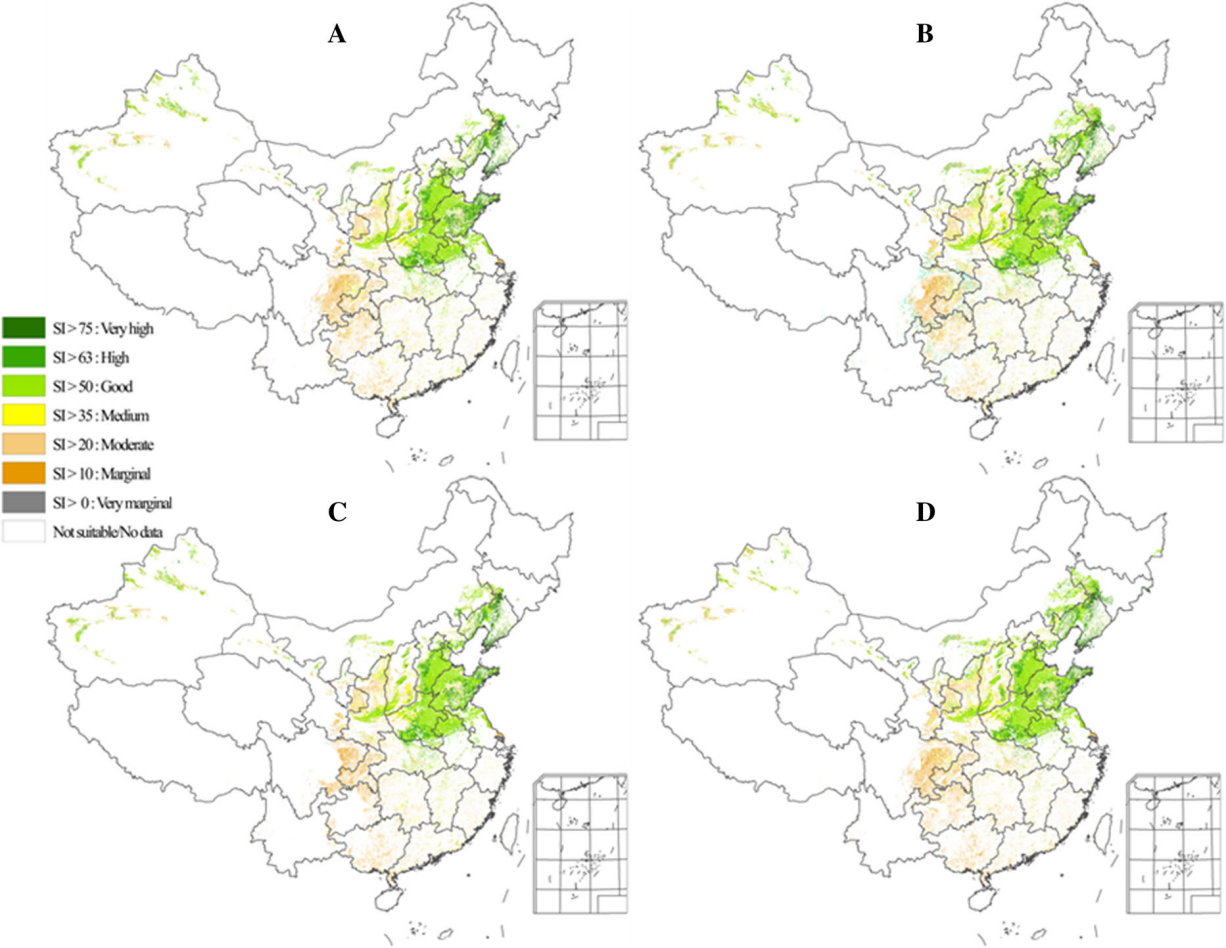
The ensemble geographical distributions of suitable areas for CMY production under the climate scenarios in the 2050s (**a** RCP2.6, **b** RCP4.5, **c** RCP6.0, and **d** RCP8.5)

Figure 5 presents the ensemble geographical distribution of suitable areas for CMY production under future climate scenarios in the 2050s (2041–2060). A comparison of Fig. 5 and Fig. 3 shows that the northern borderline of the suitable planting areas of CMY shifts northward by a significant margin. As a result, the areas belonging to the high and very high suitability classes in Liaoning, Jilin, and Inner Mongolia provinces have increased significantly, and the suitable planting areas in Shanxi and Shaanxi provinces extend significantly northward. In Shandong peninsula, the area belonging to the high and very high suitability classes for CMY cultivation has increased significantly as well. These results indicate that climate change by the 2050s will lead to a significant increase in suitable areas for CMY and will make higher latitudes suitable for CMY cultivation.

### Impact of climate change on potential yields of CMY

**Fig. 6:**
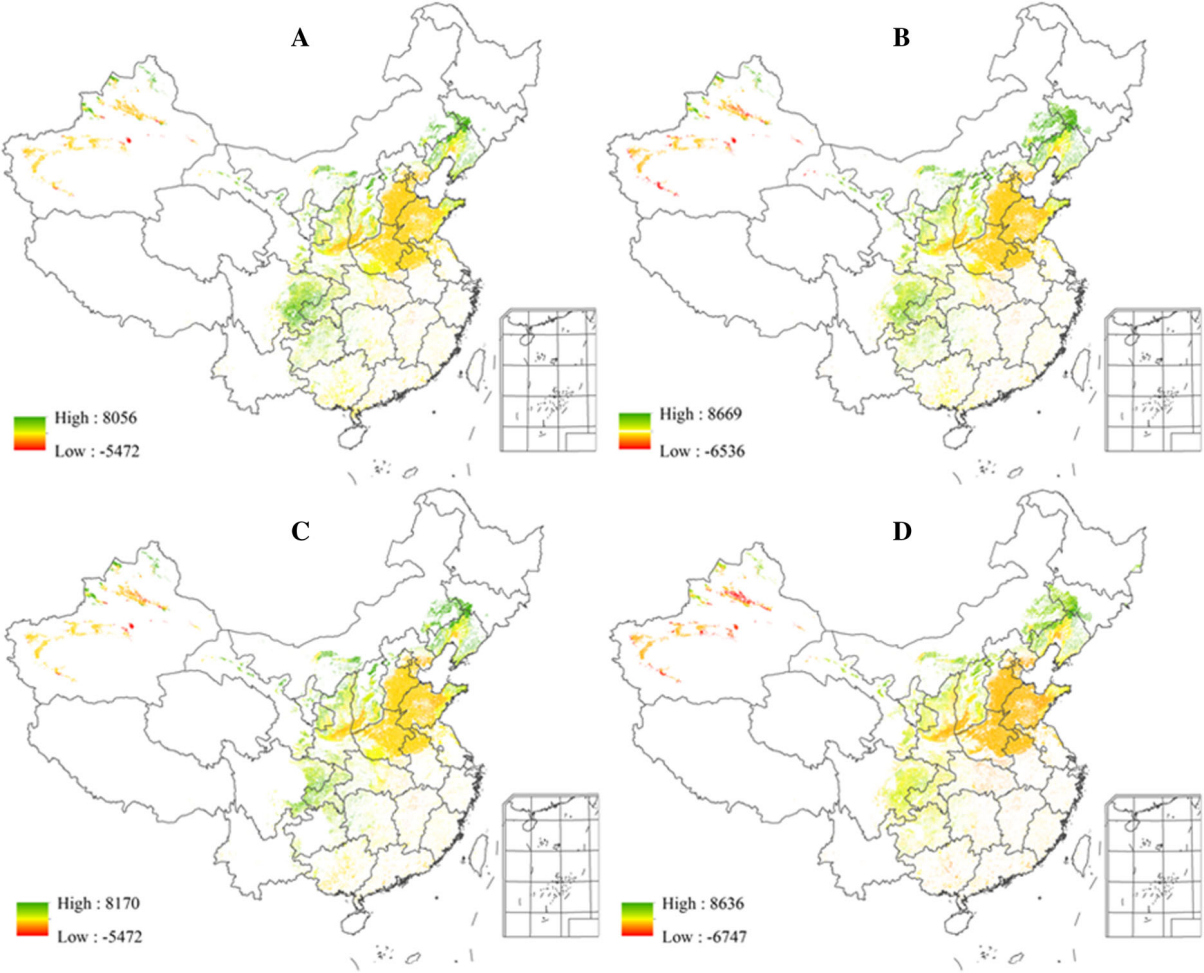
The change of CMY potential yield (kg DM/ha) between climate scenarios in the 2050s and the baseline climate in the suitable planting regions (**a** RCP2.6, **b** RCP4.5, **c** RCP6.0, and **d** RCP8.5)

Figure 6 presents the change in the potential yield (kg DM/ha) between climate scenarios in the 2050s and the baseline climate in the suitable CMY planting regions. The potential yields in the future are presented as the average over the five GCMs under each of the four future climate scenarios (i.e., RCP2.6/4.5/6.0/8.5). Figure 6 shows a clear difference between the east and the west part of the study region. The extent of yield changes is positively enhanced from south to north and from north to west, and yield increases significantly in the northern coastal areas. By the 2050s, the CMY yield in Shandong peninsula, Liaoning Province, and most of the each part of the study region would increase by a significant margin. In contrast, the yield of some of the western region such as central Shaanxi and southern Shanxi would decrease. The yield in the eastern part of Henan may decrease as well. The results in Fig. 6 also indicate the tendency of the change in CMY suitability classes. This is because in the AEZ model, the agro-ecological suitability is classified according to the level of land productivity, meaning that the change in suitability results from the change in yield. In the same grid cell, the higher the yield, the higher the suitability, the two are positively correlated.

### Aggregate results on the changes of suitable areas under future climate change scenarios

**Fig. 7:**
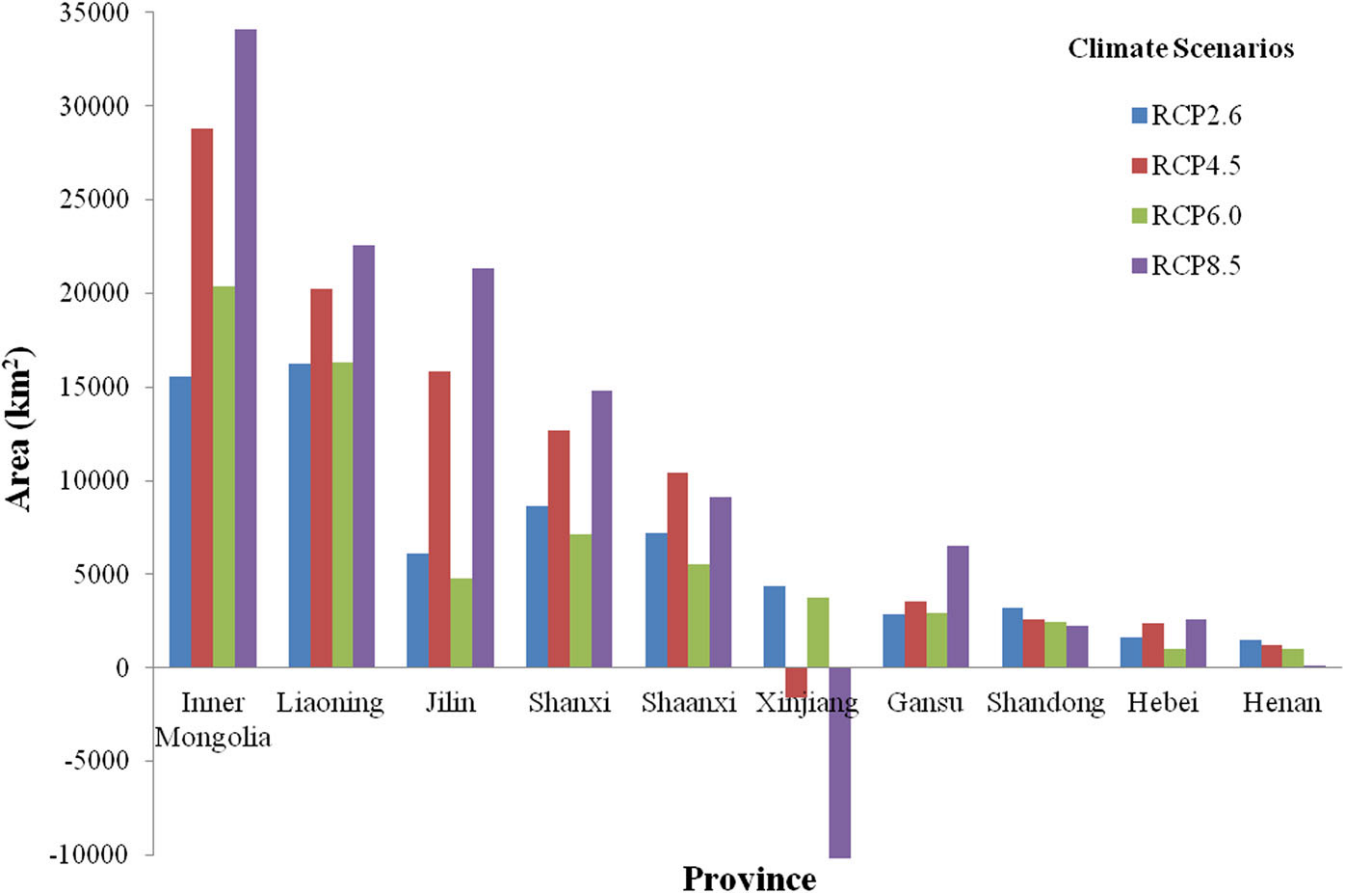
Changes in the suitable producing areas of CMY in each producing province

In order to show the aggregate trends of change in suitable producing areas under future climate change scenarios, we focus on the areas that possess more than 50% agro-ecological similarity with genuine CMY producing areas and then conduct statistical analysis for each province and for each of the 4 RCP scenarios. We obtain the value of the change by subtracting the suitable planting areas under the historical climate from the average of suitable planting areas over the five climate models for each given RCP scenario in the 2050s. The results are shown in Fig. 7. A positive value in Fig. 7 indicates that the suitable planting area for CMY in the province is increased, and the agro-ecological suitability for CMY is enhanced. A negative value indicates the opposite. Figure 7 shows that in comparison with historical climatic conditions, the area belonging to the good, high, and very high suitability classes in Inner Mongolia, Liaoning, Jilin, Shanxi, Shaanxi, and Gansu increased significantly. It can be seen that under the RCP4.5 and RCP8.5 climate scenarios, the area increase is much more significant than in the RCP2.6 and RCP6.0 scenarios. Given the fact that cropland in these provinces is less scarce, the future agricultural planting structure can be appropriately adjusted to ensure the sustainable development of the CMY industry. By contrast, the suitable producing areas in Xinjiang Province may suffer a significant decrease under the RCP4.5 and RCP8.5 scenarios by the 2050s, relative to the historical climatic conditions.

## Discussion and Conclusion

Yams are the fifth most important root crop after sweet potatoes (*Ipomoea batatas* L.) in the world and the second most important root crop in Africa after cassava (*Manihot esculenta* L.) (Srivastava et al. 2016). The production of yams has been regarded as vital for food security in Africa and other developing regions. As a result of this recognition, existing studies have mainly focused on the production quantity of yams and the impacts of climate change on production quantity (Adewuyi et al. 2014; Marcos et al. 2011; Sonder et al. 2010; Srivastava et al. 2012a, b, 2016; Zakari et al. 2014). There is a shortage of literature assessing the impacts of climate change on medical quality of Chinese Medicinal Yam (CMY). Hu et al. (2018a, b) assessed the impact of climate change on suitable planting areas for CMY based on GIS technique and similarity comparison of thirteen ecological indicators. However, their indicators are aggregate in nature because they are selected on the basis of literature review and experts’ opinions and their index-based similarity comparisons do not involve rigorous crop growth modeling at all.

To identify suitable planting area for CMY in the near future in a rigorous way and to overcome the weaknesses of the existing yam growth models in terms of lacking calibrations with modern cultivars across agro-climatic zones (Raymundo et al. 2014), in this study we collected 30-year records of CMY varieties and their corresponding phenology and agro-meteorological observations at five sites which have been regarded as the places for growing the highest quality CMY. We used these data to enrich and update the eco-physiological parameters of CMY in the agro-ecological zone (AEZ) model. We validated the updated CMY varieties and AEZ model using the publicly available production-based data under observed climate conditions. The performance of the updated AEZ-CMY in validation is much more accurate and rigorous in comparison with that of indicators-based matching in Hu et al. (2018a, b): All 42 sites in our survey match well with the suitable planting areas of CMY produced by the AEZ-CMY validation run under the baseline climate. By contrast, about 1/3 of the 42 sites in the validation run of Hu et al. (2018a) are either outside or on the borders of the suitable planting areas of CMY produced by their indicator-matching method.

After the successful validation, we employed the updated AEZ model to identify the suitable planting regions under future climate projections in China. By comparing the suitable planting areas as well as production potential under historical and future climatic conditions, we can scientifically evaluate the changes in suitable planting areas for CMY in the future. Such assessment is important for ensuring the quality and supply quantity of CMY, guiding the layout of CMY industry, and safeguarding the sustainable development of CMY resources for public health in the future.

This study has shown that regions with high agro-ecological similarity to the genuine production areas of CMY mainly locate in the lower reaches of the Yellow River and its impact plain associated with the Huaihe and Haihe river basins, including eastern Henan, southeastern Hebei, western Shandong, southern Shanxi, and central Shaanxi. It is mainly distributed in the flat land with special local soil, which is rich in many specific minerals, loose, and easy to cultivate, and seems to be the most important determinants for CMY growth in these regions. In general, the warmer climate would accelerate CMY growth, and the climate suitability of these areas will be improved due to climate change in the coming decades. As a result, these regions will continue to be the most suitable CMY planting regions and will become more productive in the future. The agro-ecological suitability of the region in northern Shaanxi, eastern Shandong, and eastern Hebei will be improved as well. This is a very good news for the CMY industry. On the other hand, new CMY varieties should be developed to minimize the negative effects of climate change on CMY production in the traditional planting areas.

There are many additional ecological factors, such as trace elements, micronutrients, and soil microorganisms, which may be important but were not included in this study. In addition, further research is needed to better understand which factors during the cultivation and field management process of CMY growing determine the quality of the medicinal components, which factors potentially increase or decrease the medicinal properties and the pharmacological effects of the plant. Further research is also needed to select attributes from the full range of geographical site characteristics of current planting areas in relation to the specific biological requirements of CMY.

## Footnotes

1 The websites we visited include Chinese Medicine Network at https://www.yt1998.com/, Chinese Herbal Medicine World at https://www.zyctd.com/, Chinese Herbal Medicine Markets at https://www.zgycsc.com/, Chinese Yam Network at https://www.zgsyw.cc/ and Honesty and Intelligence of Chinese Medicinal Materials at https://www.zyccst.com. Of these websites, https://www.zyccst.com is the only e-commerce demonstration platform of the pharmaceutical industry under the supervision by the Ministry of Commerce of China.
